# Becoming a physician for older patients: exploring the professional identity formation of medical students during a nursing home clerkship. A qualitative study

**DOI:** 10.1186/s12909-023-04835-8

**Published:** 2023-11-07

**Authors:** Annemarie Moll-Jongerius, Kirsten Langeveld, Esther Helmich, Tahir Masud, Anneke W.M. Kramer, Wilco P. Achterberg

**Affiliations:** 1 Department of Public Health and Primary Care, Leiden University Medical Center, Hippocratespad 21, Leiden, 2333 ZD The Netherlands; 2 Amsta Health Care Organization, Amsterdam, The Netherlands; 3 Department of Health Care for Older People (HCOP), Queen’s Medical Centre, Nottingham University Hospitals NHS Trust, Nottingham, UK; 4 Department of Geriatric Medicine, Odense University Hospital, Odense, Denmark

**Keywords:** Professional identity formation, Community of practice, Medical students, Older persons, Nursing home, Clerkship

## Abstract

**Background:**

To prepare medical students for the growing population of older patients, an appropriate professional identity formation is desirable. The community of practice of medical school is primarily hospital-based and disease-oriented which will lead to the development of a physician who is mainly focused on cure. This focus alone however is not always appropriate for older persons’ health care. The aim of this study is to explore the influence of participating in a nursing home community of practice on the professional identity formation of medical students.

**Methods:**

A qualitative study based on a constructivist research paradigm was conducted, using individual semi-structured, in-depth interviews and a visual narrative method (drawing) as a prompt. Thematic analysis was applied to structure and interpret the data. The study population consisted of fifth-year medical students participating in a six-week nursing home clerkship. Thirteen participants were purposefully sampled. The clerkship took place in nursing homes in the South-West of the Netherlands.

**Results:**

The medical students described the nursing home as the living environment of the patients. Actively participating in the patients’ care and experiencing the daily life of the patients was meaningful for the physician the students want to become in five ways: (1) a physician with a complete picture; (2) a physician who is close; (3) a physician who is in dialogue; (4) a physician who is able to let go and (5) a physican who collaborates.

**Conclusions:**

Caring for older patients in the nursing home influences the professional identity formation of medical students. Patient-centeredness, personal, holistic and tailored care, approachability and collaboration are important characteristics in becoming a physician for older persons’ health care. The context of this care provides relevant learning experiences for this development and the becoming of a physician in general.

## Introduction

Health concerns of older persons differ from those of younger persons and are characterized by, among other things, complexity, multimorbidity, chronic illnesses and increasing dependency [1–3]. With the growing population of older patients, future physicians will need to deal with these health challenges of older age regardless of the medical specialties they choose for their career. Therefore, medical schools have to ensure that all medical students are well prepared for older persons’ health care [4–7]. It is known that medical students feel uncomfortable with the care of older persons and consider geriatric medicine as overwhelmingly complex [8, 9]. Established geriatric competencies can be utilized in medical education to prepare medical students for geriatric care that is fit for purpose [10, 11]. Becoming a physician however goes beyond building competencies and also requires the development of a professional identity [12–15]. Therefore medical students need to develop a professional identity that enables them to provide older persons the health care they need [16].

Professional identity formation (PIF) of the medical student is related to the question ‘who do I want to become as a physician?’ and is a relatively new concept in geriatric medical education [13, 17, 18]. In the theoretical framework of socialisation PIF is seen as the process of internalizing the characteristics, values and norms of the medical profession, gradually resulting in thinking, acting, and feeling like a physician [13, 14, 19]. This process is mainly influenced by participation in the community of practice (CoP) of medical care in which engaging in patient care, observation of role models and experiences with patients are important influencers [12–14, 20–22].

A CoP is characterized by a group of professionals with shared values, knowledge base and practices [20, 23]. During medical school medical students participate in different CoPs like, among others, educational groups, hospital departments, public health facilities, primary care practices and nursing homes. It is known however that medical school is primarily hospital-based and disease-oriented with a strong focus on evidence and skills to diagnose and solve clinical problems, which will lead to development of a physician who is mainly focused on cure [16, 24–27]. This focus alone is not always appropriate for older persons’ health care, which more often requires emphasis on improving quality of life, relieving suffering and maintaining autonomy rather than on cure [26, 28, 29]. The CoP of the nursing home is described as a suitable place for medical students’ geriatric learning [30, 31]. Participating in this community can help medical students develop a professional identity that is appropriate for the care of older persons.

In a recent review we explored the literature on PIF of medical students and geriatrics [18]. It describes caring and compassion, patient-centeredness, collaboration and giving holistic and personal care as characteristics of the physician who takes care of older persons. Role models, building relationships with older persons and participating in their lives are mentioned as important influencers of PIF. The studies in this review however are particularly based on one day or preclinical experiences of medical students with older patients.

In this study we further explore the PIF of medical students in relation to older persons’ health care. A deeper understanding can help medical educators to guide medical students in developing an appropriate professional identity for this care. To this end we use experiences of fifth year clinical medical students during a six week clerkship in a nursing home. The aim is to gain more insight into the influence of such a context on their PIF. Our research questions are:

1. What perceptions do medical students have of the physician they want to become after participating in the nursing home CoP?
2. What experiences during the nursing home clerkship had impact on these perceptions?

## Methods

### Methodology

We conducted an explorative, qualitative study based on a constructivist research paradigm. In this philosophical framework reality can only be understood through people’s experiences and interpretations [32]. We used the meaningful experiences of medical students while they participated in the CoP of the nursing home, to better understand the influence of caring for older patients on the physican they want to become. We applied thematic analysis to structure and interpret the data [33–35].

### Research team

Our research team included an elderly care physician, medical teacher and PhD candidate in medical education (AM), a cultural/medical anthropologist, medical teacher and qualitative researcher (KL), an elderly care physician and qualitative researcher (EH), a geriatrician and professor in geriatric medicine (TM), a general practitioner and professor in medical education (AK), and an elderly care physician and professor of institutional care and elderly care medicine (WA).

### Context

We conducted this study between June and December 2018 at the Leiden University Medical Center in the Netherlands, where fifth-year medical students participate in a six-week mandatory clerkship in a nursing home. Prior to this clerkship, students completed all clinical clerkships in hospital. After the clerkship, they entered the last year of medical school, consisting of elective courses.

The nursing home clerkship took place in nursing homes in the South-West of the Netherlands. Dutch nursing homes provide psychogeriatric, long-term somatic care and rehabilitation care for older patients who cannot live at home. The main difference with other countries is that in the Netherlands medical care in the nursing home is delivered by a specifically trained nursing home physician; an ‘elderly care physician’ [36]. This physician is employed by the nursing home and works together in a multidisciplinary team with other health professionals; psychologists, speech therapists, dieticians, occupational therapists, physiotherapists and spiritual counsellors, who are also employed by the nursing home. During the nursing home clerkship medical students are supervised by the nursing home physician and they are part of the multidisciplinary team.

### Participants

Our study population consisted of medical students entering the nursing home clerkship. Two weeks before the start of the clerkship, students were invited to participate by AM during a lecture at the university. AM provided information about the study and asked the students to reply by email if they wanted to participate. Every four weeks a new group of students started with the clerkship and AM continued to invite students until the research team felt data saturation had been achieved [37]. To ensure a rich diversity within the data and a representative sample, we sampled purposefully to select medical students who differed in gender, age, work experience, social background, cultural and/or religious backgrounds. Men were approached explicitly to try and improve the gender balance.

### Data collection

As our goal was to understand the students’ experiences, we used individual semi-structured, in-depth interviews and a visual narrative method (drawing) as a prompt. Drawing as a pre-interview activity can help to narrate the meaning of experiences more deeply [38–40]. Participants were invited for two interview sessions with AM, one session one week before the nursing home clerkship and one session in the last week of this clerkship. The pre-clerkship session took place at the university, and the end-of-clerkship session in the nursing home. First, the students were asked to make a drawing in response to the question *‘who do I want to become as a physican’*. They made this drawing alone in a separate room, taking as much time as they needed. After finishing the drawing AM conducted an individual, semi-structured, in-depth interview in which the students shared what was important to them when they answer the question ‘*who do I want to become as a physician’*. Their drawing was used as a prompt, to help the students reflect on the topic. The same procedure was followed after the clerkship. The students received their pre-clerkship drawing and were asked to supplement it, leave it unchanged or make a new drawing in response to the same question *‘who do I want to become as a physician’*. AM then conducted an individual, semi-structured, in-depth interview in which they shared and reflected on the perception of the physician they want to become after the nursing home clerkship and what experiences in the nursing home had contributed to this, using both drawings as a prompt. Drawing took approximately 30 min. The interviews lasted approximately one hour and were audio recorded and transcribed verbatim. We carried out a linguistic transcription, not phonetic but orthographic [41].

### Data analysis

Following the standard steps of thematic analysis we first analyzed the pre-clerkship interviews to identify a set of major themes and subthemes, using open coding and an iterative analysis [34, 35]. The drawings, used only as a prompt for the students to reflect on the topic during the interview, were not analyzed as a separate data set. For the coding process, the first researcher AM reviewed the first two interviews by reading and re-reading the transcripts, listening to the audio tapes, and constructing a list of open codes. The senior researcher (KL) independently reviewed those two interviews by reading and re-reading the transcripts. AM and KL discussed the open codes and organized them into a list of starting codes. Then three more interviews were reviewed in the same way. AM and KL discussed new codes that emerged and inductively redefined the codes into a coding scheme, generating themes related to the research question. Subsequently AM analyzed another two interviews by applying the coding scheme and discussing evolving themes with KL and AK. This iterative process was repeated until no new themes emerged. The coding scheme was finalized after the analysis of nine interviews, and approved by the research team. Four additional interviews did not add new ideas. The final themes were grouped into an overview of major themes and subthemes to capture the essence of what is important to the students when answering the question ‘*who do I want to become as a physician’*.

After the analysis of all pre-clerkship interviews, the post-clerkship transcripts were analyzed in the same way, using the pre-clerkship themes as a comparison. To capture the influence of caring for older patients in the nursing home CoP on medical students’ PIF, changes in pre-clerkship themes, new themes, reinforcement or confirmation of pre-clerkship themes were added in the final post-clerkship overview of major themes. Together with the research team AM and KL gave meaning to each theme and created narrative descriptions to explain the broader stories each theme tells. These stories are described in the results, using quotes of participants to illustrate key features [34, 35]. AM kept a journal throughout the process of data-gathering and analysis in which she recorded field notes and reflected on the choices she and the research team made.

### Ethics

This project was approved by the Ethical Research Board of the Netherlands Association of Medical Education (NVMO-ERB, no 1056). Participation was fully voluntary and confidential, also to ensure that it would not have consequences for students’ assessments. The participants provided written informed consent, part of which was consent to publish anonymized responses. Only AM knew the participants’ identities and listened to the audio recordings. To guarantee confidentiality, each participant was given a number. The researchers were not involved in any teaching activities or assessments of the students.

## Results

Thirteen fifth-year medical students (ten female) participated in this study. They described the nursing home as the living environment of the patients in which they could work as ward physicians under supervision. By doing so they actively participated in the patients’ care, resulting in *‘truly experiencing’* the daily life of the patients, up close and over a longer period of time. The students mentioned that this helped them *‘really get to know’* the patient as a ‘*person’*. Student 4 shared:

Yes, just looking at it differently. Less like, well, an object. Literally…try to always remember…that there is a complete human being in front of me.

This experience was meaningful for the physician they want to become in five ways: [1] a physician with a complete picture; [2] a physician who is close; [3] a physician who is in dialogue; [4] a physician who is able to let go and [5] a physician who collaborates.

### A physician with a complete picture

Many students related to the *‘complete picture’*, meaning the whole life of the patient like *‘family, background, interests and emotions’*. They described that, as compared to the hospital, this complete picture received *‘much more attention’* from the health care professionals. Student 5 shared what was important to her, based on a patient’s discharge:

But here we always think about what happens afterwards…I think that’s very important…the occupational therapist converts the whole house when people go home… Just a lot more looking at the big picture.

**Fig. 1:**
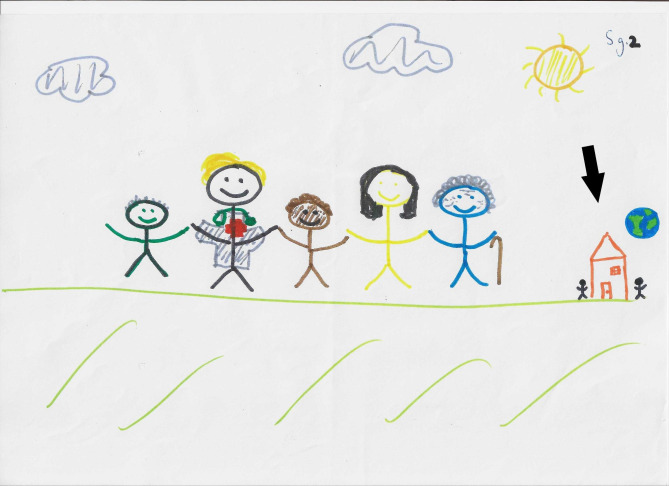
Student 9 added a house and a globe (see black arrow) to his post clerkship drawing and explained this meant the attention to the whole life of the individual patient

Student 9 added a house and a globe to his post clerkship drawing and explained this meant the attention to the whole life of the individual patient. (Fig. 1)

Some students perceived the consequences of being ill or a treatment on this whole life. Student 13 shared her experience with a patient who was treated with diuretics:

I never realized that…it just has a lot more impact than you actually envision…Until you suddenly see it up close. That someone needs to go to the bathroom all the time, but this is actually not possible with those legs…I think you often overlook that….

Students experienced they could better help the patients if the care takes into account this complete picture and not just a *‘small part’* of it. This way of caring confirmed their desire to become a physician who tailors the care to the patient’s whole life.

### A physician who is close

**Fig. 2:**
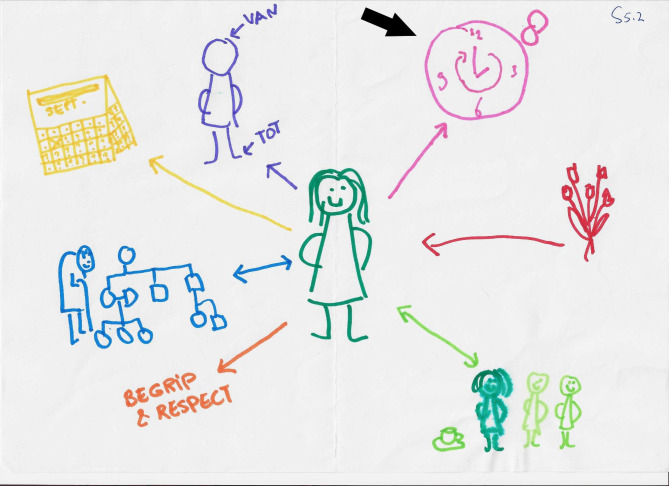
Student 5 added a clock (see black arrow) to her post clerkship drawing and explained this meant ‘taking time’ for the patient

Several students described they could give the patient personal attention by *‘little things’* such as *‘a chat’* or *‘drinking a cup of coffee’*. Student 5 added a clock to her post clerkship drawing and explained this meant *‘taking time’* for the patient. (Fig. 2) As a result, they experienced closeness to the individual patient. The closeness of the supervisor with a *‘human’* and *‘approachable’* interaction with the patient, was mentioned as an example for their own physician patient interaction. Student 4 expressed:

The atmosphere and the setting and the time…You do learn to be very person-to-person…not physician-to-…It’s all very low-threshold.

They also expressed that closeness could be communicated by not wearing a white coat or through a touch. Student 13 experienced her supervisor as a role model:

…how you can sit down next to a patient, and put an arm around someone…that was so beautiful. That I really thought: that’s what it’s all about, this is really who you are as a physician.

Students experienced that patients *‘opened up more’* when they were close to the patient and they realized that human closeness is important for the physician they want to become.

### A physician who is in dialogue

Several students described that the nursing home physician made treatment decisions together with the patients and it was normal to be *‘next to the patient’* without *‘hierarchy’*. Most students were present during the conversations between the physician and the patient and described that it was important for the physician to *‘accommodate’* to the patient and to *‘respect wishes’*. Student 4 described the physician as less determinative and the relationship with the patient as human:

*That you really are not so much the leader, but you really do it in dialogue with the patient*…*Provide advice and support. Less dictating…I’m not sure how to describe it…just more one human being to another.*

These experiences confirmed the students they want to become a physician who makes treatment decisions together with the patient. Student 2 explained:

…always realized it, but maybe a little more so now…part of the patient is also what they want or don’t want anymore…and that you also give that more consideration in your decision. That, of course, is most important.

### A physician who is able to let go

Most students experienced patients who could not be cured up close. They observed that continuing curative treatment was very stressful for the patients. This had an impact on them. Student 3 shared her experience:

That Parkinson’s patient, no more treatment options, we had arranged an admission…and that morning he said ‘I want to die’… so maybe you also recognize, yes, it is very burdensome. And you don’t always realize that, that you understand better.

Students were impressed by the nursing home physician who was able to *‘let go of’* the curative treatment and doing *‘what is best at that moment’*. To the students, abandoning curative treatment seemed very difficult. They described it as a *‘new experience’* and emphasized they want to become a physician who asks the question whether he should *‘put a person through this treatment*’. Student 4 expressed that knowing the patient as a *‘human being’* was helpful to her:

So yes, it’s different…it’s about a human being, there’s a human being in front of you. Rather than treat, treat, treat, it’s more stop or do nothing.

### A physician who collaborates

Several students described their experience of being part of a multidisciplinary team. They experienced that *‘with different views you can achieve more’*, which benefits the care for the patient. Some students realized that a physician is ‘*really dependent on others*’ and *‘cannot do it on his own’*. Student 3 shared:

I also realized that yes, how small you actually are…when you work in the hospital it’s more heroic…we’re going to help people…but…you really couldn’t do anything without that collaboration…you really are depended on the team.

Students described that, as compared to the hospital, working together with other health professionals was *‘natural, low-treshold and without hierarchy’*. Students experienced this way of working in a team as *‘for the first time*’ and important for the physician they want to become. Student 9 shared:

So I have learned a complete different form of collaboration here…And that is something I really take with me.

## Discussion

In this study we explore the PIF of medical students in relation to the care of older persons. To our knowledge this is the first study in which clinical medical students are specifically asked about becoming a physician after participating in the nursing home CoP over a longer period of time. This participation influences the perceptions of becoming a physician. Caring of older patients reinforces and confirms the students to become a physician who tailors the care to the patients’ whole life, makes treatment decisions together with the patient and is close to the patient. Furthermore, it creates the intention to become a physician who is able to let go of curative treatment and who works together in a team with other health professionals.

These findings broaden our understanding of PIF of medical students in relation to geriatrics. To develop an appropriate professional identity, medical students need to become patient-centered, collaborative, and able to give holistic and personal care [18]. Our results emphasize these characteristics and add new ones, creating a more complete picture of this professional identity. First it is essential to become a physician who is able to give tailored care by avoiding unnecessary curative treatment. Furthermore the older patient needs a physician who takes time and is approachable. Finally a non-hierarchical attitude to both the patient and other health care professionals facilitates collaboration and dialogue. This picture of characteristics has similarities with the concept of ‘whole person care’ that is used to describe holistic care in nursing and general practice [42–46].

Several experiences in the nursing home CoP support the students’ becoming. Our study underlines engaging in patient care, observation of role models and experiences with patients as influencers of PIF [12–14, 20–22]. In addition the findings make explicit which elements are essential to the process of PIF in relation to the care of older persons. Due to the participation in a multidisciplinary team other health care professionals can contribute to this process by being role models. This participation also encourages the development of a holistic approach [47]. Furthermore, being part of the living environment and lives of the older person over a longer period of time creates the opportunity to build a physician patient relationship. This results in a better understanding of patients’ needs and well being. It is known that having long term relationships with patients influences PIF and stimulates the development of personal care, patient-centeredness, humanity and compassion [48, 49].

### Implications for medical education

Based on our findings we have two considerations for medical education. First, to become a physician of older persons, participation in an appropriate CoP is essential. This CoP has to provide role models and practices that represent the values and norms relevant to this care [12–14, 20–23]. The nursing home and the care of older patients at home are described as suitable communities [30, 31, 50, 51]. These contexts can also supplement the dominant hospital CoP characterised by disease centredness and a more hierarchical relationship between physician and patient or other health professionals [25–27, 52, 53]. Additionaly, the values of patient-centeredness, personal and holistic care, approachability and collaboration are not only important to older persons’ health care but also to the care of all patients. This suggests that participation in the CoP of caring of older persons is also relevant to the becoming of a physician in general [16, 18, 54, 55]. Therefore curriculum committees should be aware of the value of the learning opportunities of this community.

Second, we want to emphasize the importance of building long term relationships with older persons. By being part of the personal life medical students learn to know the older patient as a person and will better understand the needs and expectations of the older patient. Longitudinal integrated clerkships in general practice or educational projects that facilitate long term contacts with community living older adults can stimulate and facilitate these relationships [48, 56].

### Strenghts and limitations

Our study has strengths and limitations. A strength of the study was the use of in-depth interviews with a drawing as a prompt. Taking time and the visual narrative method as pre-interview activity offered the students different ways to share their experiences and thoughts [38, 40]. Furthermore, AM is an elderly care physician and understood the context of the nursing home. This may have created safety and rapport during the interviews. Conversely, being an elderly care physician may have influenced the interpretation of the data which is a limitation. To ensure reflexivity, the data were analyzed together with a cultural/medical anthropologist and a general practitioner, and AM kept a journal. The participation of another health care professional, like a occupational therapist, nurse or psychologist in the research team could have enriched the analyses of the data.

The period of participating in the nursing home was six weeks. This period was longer than in previous studies, however, the long term effects on PIF are not known which limit our conclusions. Furthermore we used two research methods, interviewing and drawing, to explore the experiences of the students. Triangulation through, for example, observation would have further deepened our understanding. The sample size of medical students from one Dutch university may limit the generalizability of our findings to medical schools outside the Netherlands. The small amount of men in the sample is almost representative for Dutch medical schools. Finally, we realize that the specific context of Dutch nursing homes with specialized nursing home physicians and medical care is uncommon in the world. We believe however that this unique situation can contribute to enhancement of (geriatric) medical education internationally.

### Future research

Given the growing population of older patients and the need to prepare medical students for this care with an appropriate PIF, more research is needed to further explore this development. A future research area will be the perspectives of older persons and physicians on the development of a professional identity and older persons’ healthcare. Furthermore the understanding and knowledge of PIF has been evolved over the years. New developed conceptual models can help us to further explore this process [57]. In the end we intend to develop educational interventions to encourage this PIF based on the literature and our findings. A better understanding can facilitate medical educators to help medical students develop a professional identity that enables them to give older patients the health care they need.

## Conclusion

To prepare medical students for the growing population of older patients, an appropriate PIF is desirable. In this study, we explored the influence of the CoP of the nursing home on the PIF of medical students. The aim was to gain more insight in the development of an appropriate professional identity for older persons’ health care. To our knowledge this is the first study in which clinical medical students are specifically asked about their perception of becoming a physician, after caring for older patients over a longer period of time. Patient-centeredness, personal, holistic and tailored care, approachability and collaboration are important characteristics in this becoming. The context of older persons’ health care provides relevant learning experiences for the development of an appropriate professional identity for the care of older persons and for the becoming of a physician in general.

## Data Availability

The datasets generated and analysed during this study are not publicly available due to promised anonymity of the participants, but are available from the corresponding author on reasonable request and with permission of the participants in question.

## Abbreviations

CoP: Community of Practice
PIF: Professional Identity Formation

## References

[1] Abdi S, Spann A, Borilovic J, de Witte L, Hawley M (2019). Understanding the care and support needs of older people: a scoping review and categorisation using the WHO international classification of functioning, disability and health framework (ICF). BMC Geriatr.

[2] Banerjee S (2015). Multimorbidity–older adults need health care that can count past one. Lancet.

[3] Limpawattana P, Phungoen P, Mitsungnern T, Laosuangkoon W, Tansangworn N (2016). Atypical presentations of older adults at the emergency department and associated factors. Arch Gerontol Geriatr.

[4] Tullo ES, Spencer J, Allan L (2010). Systematic review: helping the young to understand the old. Teaching interventions in geriatrics to improve the knowledge, skills, and attitudes of undergraduate medical students. J Am Geriatr Soc.

[5] Oakley R, Pattinson J, Goldberg S, Daunt L, Samra R, Masud T (2014). Equipping tomorrow’s doctors for the patients of today. Age Ageing.

[6] Leipzig RM, Granville L, Simpson D, Anderson MB, Sauvigné K, Soriano RP (2009). Keeping granny safe on July 1: a consensus on minimum geriatrics competencies for graduating medical students. Acad Med.

[7] Pershing S, Fuchs VR. Restructuring medical education to meet current and future health care needs 2013 [updated Dec. 2013/10/17:[1798 – 801]. 10.1097/ACM.0000000000000020 24128642

[8] Meiboom AA, de Vries H, Hertogh CM, Scheele F (2015). Why medical students do not choose a career in geriatrics: a systematic review. BMC Med Educ.

[9] Bagri AS, Tiberius R (2010). Medical student perspectives on geriatrics and geriatric education. J Am Geriatr Soc.

[10] Core competencies for the care of older patients: recommendations of the American Geriatrics Society (2000). The Education Committee Writing Group of the American Geriatrics Society. Acad Med.

[11] Masud T, Blundell A, Gordon AL, Mulpeter K, Roller R, Singler K (2014). European undergraduate curriculum in geriatric medicine developed using an international modified Delphi technique. Age Ageing.

[12] Jarvis-Selinger S, Pratt DD, Regehr G (2012). Competency is not enough: integrating identity formation into the medical education discourse. Acad Med.

[13] Cruess RL, Cruess SR, Boudreau JD, Snell L, Steinert Y (2014). Reframing medical education to support professional identity formation. Acad Med.

[14] Monrouxe LV (2010). Identity, identification and medical education: why should we care?. Med Educ.

[15] Cruess SR, Cruess RL, Steinert Y (2019). Supporting the development of a professional identity: General principles. Med Teach.

[16] van de Pol MHJ, Lagro J, Koopman EL, Olde Rikkert MGM, Fluit C, Lagro-Janssen ALM (2018). Lessons learned from narrative feedback of students on a geriatric training program. Gerontol Geriatr Educ.

[17] Helmich E, Yeh HM, Kalet A, Al-Eraky M (2017). Becoming a doctor in different cultures: toward a Cross-cultural Approach to supporting professional identity formation in Medicine. Acad Med.

[18] Moll-Jongerius A, Langeveld K, Tong W, Masud T, Kramer AWM, Achterberg WP. Professional identity formation of medical students in relation to the care of older persons: a review of the literature. Gerontol Geriatr Educ. 2023:1–14. 10.1080/02701960.2023.2210559 37170948

[19] Irby DM, Hamstra SJ (2016). Parting the clouds: three professionalism frameworks in Medical Education. Acad Med.

[20] Cruess RL, Cruess SR, Steinert Y (2018). Medicine as a community of practice: implications for Medical Education. Acad Med.

[21] Cruess RL, Cruess SR, Boudreau JD, Snell L, Steinert Y (2015). A schematic representation of the professional identity formation and Socialization of medical students and residents: a guide for medical educators. Acad Med.

[22] Jarvis-Selinger S, MacNeil KA, Costello GRL, Lee K, Holmes CL (2019). Understanding professional identity formation in early clerkship: a Novel Framework. Acad Med.

[23] Lave J, Wenger E. Situated learning: legitimate peripheral participation. Cambridge University Press; 1991.

[24] Monrouxe L. Negotiating professional identities: Dominant and contesting narratives in medical students’ longitudinal audio diaries. Curr Narratives. 2009;1.

[25] MacLeod A (2011). Caring, competence and professional identities in medical education. Adv Health Sci Educ Theory Pract.

[26] Longino CF (1997). Jr. Pressure from our aging population will broaden our understanding of medicine. Acad Med.

[27] Fox E (1997). Predominance of the curative model of medical care. A residual problem. JAMA.

[28] Franco AA, Bouma H, Bronswijk JEMHV. Health care paradigms in transition. Gerontechnology. 2014;13(1).

[29] Kogan AC, Wilber K, Mosqueda L (2016). Person-centered care for older adults with chronic conditions and functional impairment: a systematic literature review. J Am Geriatr Soc.

[30] Huls M, Rooij SE, Diepstraten A, Koopmans R, Helmich E (2015). Learning to care for older patients: hospitals and nursing homes as learning environments. Med Educ.

[31] Masud T, Ogliari G, Lunt E, Blundell A, Gordon AL, Roller-Wirnsberger R et al. A scoping review of the changing landscape of geriatric medicine in undergraduate medical education: curricula, topics and teaching methods. Eur Geriatr Med.16. 10.1007/s41999-021-00595-0 PMC8720165 34973151

[32] Kahlke RM (2014). Generic qualitative approaches: pitfalls and benefits of Methodological Mixology. Int J Qualitative Methods.

[33] Bennett D, Barrett A, Helmich E (2019). How to… analyse qualitative data in different ways. Clin Teach.

[34] Braun V, Clarke V (2006). Using thematic analysis in psychology. Qualitative Res Psychol.

[35] Kiger ME, Varpio L (2020). Thematic analysis of qualitative data: AMEE Guide No. 131. Med Teach.

[36] Koopmans RT, Lavrijsen JC, Hoek JF, Went PB, Schols JM (2010). Dutch elderly care physician: a new generation of nursing home physician specialists. J Am Geriatr Soc.

[37] Hennink M, Kaiser BN (2022). Sample sizes for saturation in qualitative research: a systematic review of empirical tests. Soc Sci Med.

[38] Ellis J, Hetherington R, Lovell M, McConaghy J, Viczko M (2013). Draw me a picture, tell me a story: evoking memory and supporting analysis through pre-interview drawing activities. Alta J Educational Res.

[39] Cristancho S, Bidinosti S, Lingard L, Novick R, Ott M, Forbes T (2015). Seeing in different ways: introducing rich pictures in the study of expert judgment. Qual Health Res.

[40] Rees C (2018). Drawing on drawings: moving beyond text in health professions education research. Perspect Med Educ.

[41] Bucholtz M (2000). The politics of transcription. J Pragmat.

[42] Carter MA, Haji Assa AS (2023). The problem of comparing nurse practitioner practice with medical practice. Nurs Inq.

[43] Papathanasiou I, Sklavou M, Kourkouta L (2013). Holistic nursing care: theories and perspectives. Am J Nurs Sci.

[44] Tarrant C, Windridge K, Boulton M, Baker R, Freeman G (2003). How important is personal care in general practice?. BMJ.

[45] Strandberg EL, Ovhed I, Borgquist L, Wilhelmsson S (2007). The perceived meaning of a (w)holistic view among general practitioners and district nurses in Swedish primary care: a qualitative study. BMC Fam Pract.

[46] Thomas H, Mitchell G, Rich J, Best M (2018). Definition of whole person care in general practice in the English language literature: a systematic review. BMJ Open.

[47] Jentoft R (2021). Boundary-crossings among health students in interprofessional geropsychiatric outpatient practice: collaboration with elderly people living at home. J Interprofessional Care.

[48] Konkin J, Suddards C (2012). Creating stories to live by: caring and professional identity formation in a longitudinal integrated clerkship. Adv Health Sci Educ Theory Pract.

[49] Adams J, Ari M, Cleeves M, Gong J (2020). Reflective writing as a window on Medical Students’ Professional Identity Development in a Longitudinal Integrated Clerkship. Teach Learn Med.

[50] Helmich E, Derksen E, Prevoo M, Laan R, Bolhuis S, Koopmans R (2010). Medical students’ professional identity development in an early nursing attachment. Med Educ.

[51] Helmich E, Bolhuis S, Prins J, Laan R, Koopmans R (2011). Emotional learning of undergraduate medical students in an early nursing attachment in a hospital or nursing home. Med Teach.

[52] Engel GL (2012). The need for a new medical model: a challenge for biomedicine. Psychodyn Psychiatry.

[53] Allen D, Wainwright M, Mount B, Hutchinson T (2008). The wounding path to becoming healers: medical students’ apprenticeship experiences. Med Teach.

[54] Kanter SL (2012). The nursing home as a core site for educating residents and medical students. Acad Med.

[55] Shield RR, Farrell TW, Campbell SE, Nanda A, Wetle T (2015). Professional development and exposure to geriatrics: medical student perspectives from narrative journals. Gerontol Geriatr Educ.

[56] Goldman JS, Trommer AE (2019). A qualitative study of the impact of a Dementia experiential learning project on pre-medical students: a friend for Rachel. BMC Med Educ.

[57] Joseph ML, Edmonson C, Godfrey N, Kuhl L, Shaffer F, Owens R (2023). A conceptual model for professional identity in nursing: an interdependent perspective. Nurs Sci Q.

